# Serious complications in COVID-19 ARDS cases: pneumothorax, pneumomediastinum, subcutaneous emphysema and haemothorax

**DOI:** 10.1017/S0950268821001291

**Published:** 2021-06-08

**Authors:** Bulent Baris Guven, Tuna Erturk, Özge Kompe, Ayşın Ersoy

**Affiliations:** Department of Anesthesia and Reanimation, University of Health Sciences Turkey, Sultan 2. Abdulhamid Han Training and Research Hospital, Istanbul, Turkey

**Keywords:** ARDS, Coronavirus, COVID-19, Pneumothorax, SARS Cov-2

## Abstract

The novel coronavirus identified as severe acute respiratory syndrome-coronavirus-2 causes acute respiratory distress syndrome (ARDS). Our aim in this study is to assess the incidence of life-threatening complications like pneumothorax, haemothorax, pneumomediastinum and subcutaneous emphysema, probable risk factors and effect on mortality in coronavirus disease-2019 (COVID-19) ARDS patients treated with mechanical ventilation (MV). Data from 96 adult patients admitted to the intensive care unit with COVID-19 ARDS diagnosis from 11 March to 31 July 2020 were retrospectively assessed. A total of 75 patients abiding by the study criteria were divided into two groups as the group developing ventilator-related barotrauma (BG) (*N* = 10) and the group not developing ventilator-related barotrauma (NBG) (*N* = 65). In 10 patients (13%), barotrauma findings occurred 22 ± 3.6 days after the onset of symptoms. The mortality rate was 40% in the BG-group, while it was 29% in the NBG-group with no statistical difference identified. The BG-group had longer intensive care admission duration, duration of time in prone position and total MV duration, with higher max positive end-expiratory pressure (PEEP) levels and lower min pO_2_/FiO_2_ levels. The peak lactate dehydrogenase levels in blood were higher by statistically significant level in the BG-group (*P* < 0.05). The contribution of MV to alveolar injury caused by infection in COVID-19 ARDS patients may cause more frequent barotrauma compared to classic ARDS and this situation significantly increases the MV and intensive care admission durations of patients. In terms of reducing mortality and morbidity in these patients, MV treatment should be carefully maintained within the framework of lung-protective strategies and the studies researching barotrauma pathophysiology should be increased.

## Introduction

First emerging in Wuhan state in China, the novel coronavirus identified as severe acute respiratory syndrome-coronavirus-2 (SARS-CoV-2) has infected more than 100 million people around the world since December 2019 and continues to spread rapidly. With very rapid spread in a short duration due to high transmissivity and rapid speed, it causes severe pneumonia tableau that can progress to acute respiratory distress syndrome (ARDS) in humans. This disease, defined as coronavirus disease-2019 (COVID-19) by the World Health Organization (WHO), was accepted as a pandemic on 11 March 2020 and Turkey declared its first case on the same date [1]. Since then, our hospital has operated as a pandemic hospital with a total of 96 patients with COVID-19 diagnosis, severe pneumonia or ARDS tableau and respiratory failure admitted to the intensive care up to 31 July 2020.

A common feature of all patients admitted to intensive care is continuation of hypoxaemia in spite of high amounts of oxygen support and meeting the Berlin criteria used for diagnosis of ARDS. Accordingly, patients with respiratory distress newly occurring or worsening within 1 week, radiological bilateral pulmonary involvement unexplained by nodules or collapse, presence of hypoxaemia without a cause like cardiac disease or excessive fluid loading, and O_2_ rate in inspirium air fraction of partial arterial oxygen pressure (PaO_2_ / FiO_2_) ⩽300 mmHg were diagnosed with ARDS, while the condition of positive real-time PCR was required for COVID-19 diagnosis [2].

It is reported that barotrauma develops at rates of 6.5% during mechanical ventilation (MV) during the treatment of ARDS patients [3]. Barotrauma and high positive pressure in the lungs may cause complications like pneumothorax (PX), pneumomediastinum or subcutaneous emphysema leading to lengthened duration of intensive care admission and increased mortality of patients [3,4]. As a result, many studies were conducted regarding developing lung-protective ventilation strategies especially for ARDS patients [5, 6]. However, currently barotrauma still continues to be a serious problem encountered by ARDS patients.

The primary aim of our study is to reveal probable causes of life-threatening complications such as PX, haemothorax, pneumomediastinum and subcutaneous emphysema that may occur during invasive MV of COVID-19 patients monitored and treated in intensive care with the ARDS tableau in line with the same protocols and to retrospectively assess the effect on mortality and risk factors.

The secondary aim of the study is to determine the type, frequency and predisposing factors for barotrauma occurring in patients receiving invasive MV treatment due to SARS-CoV-2 infection and to predict complications that may develop in this way.

## Subjects and methods

The study was completed with retrospective assessment of 96 adult patients admitted to Health Sciences University, İstanbul Sultan Abdulhamid II Education and Research Hospital, Anaesthesiology and Reanimation intensive care unit developing ARDS linked to SARS-CoV-2 infection from 11 March to 31 July 2020. All patients monitored for ARDS diagnosis in our clinic had the COVID-19 ARDS monitoring and treatment protocols applied. Within the framework of this protocol, patients had SARS-CoV-2 infection diagnosis made with real-time PCR test and the test was repeated every 10–14 days. During admission to the ICU patients had thoracic high-resolution computed tomography (HRCT) images taken and bedside chest radiography was taken at 1- to 3-day intervals according to clinical requirements during treatment in intensive care. All patients admitted to intensive care were administered standard antiviral treatment for SARS-CoV-2 infection. Patients with respiratory failure in spite of high-flow oxygen therapy (HFOT) or non-invasive ventilation (NIV) treatment, with disrupted mental status, hypoxia and continuing haemodynamic instability findings had orotracheal intubation performed and were linked to a mechanical ventilator in line with lung-protective ventilator strategies. Positive end-expiratory pressure (PEEP) values were set according to the ARDSnet lower PEEP/higher FiO_2_ table [5]. Patients who responded to PEEP increase and were haemodynamically stable had the recruitment manoeuvre (RM) performed 2 times per day after neuromuscular blocker administration with actual FiO_2_ and airway pressure 30 cm H_2_O lasting 60 s in CPAP mode [7]. Patients with PaO_2_/FiO_2_ values below 150 were placed in prone position for 16–24 h duration.

**Fig. 1.**
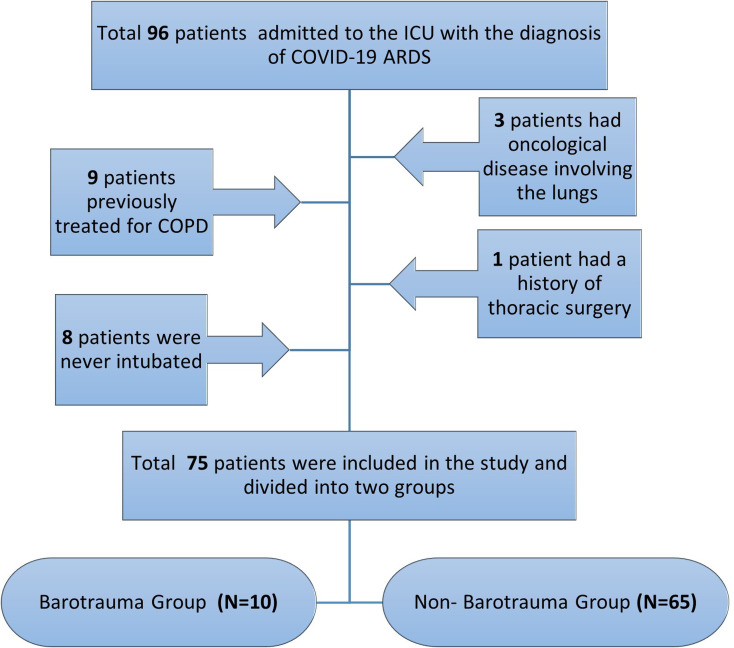
Flow chart of the retrospective study. (General distribution and barotrauma status of COVID-19 patients developing ARDS).

The aim of the study was to assess barotrauma caused by invasive MV treatment and accompanying complications in COVID-19 ARDS cases, so patients with predisposing factors for barotrauma formation were excluded from the study. As a result, cases with previous chronic obstructive pulmonary disease (COPD) treatment (*N* = 9), history of previous thoracic surgery (*N* = 1), who were not intubated (*N* = 8), with primary or metastatic lung tumours (*N* = 3), with iatrogenic (catheterisation, thoracentesis, etc.) pulmonary trauma (*N* = 0) and bullae, blebs or cysts on lung images taken on first admission to intensive care (*N* = 0) were excluded from the study. The 75 patients meeting the criteria were divided into two groups as those developing ventilator-related barotrauma (group BG) (*N* = 10) and those not developing ventilator-related barotrauma (group NBG) (*N* = 65) (shown in Fig. 1).

During collection of data, ventilator setting parameters such as peak inspiratory pressure (PIP), driving pressure (ΔP), plateau pressure (Pplt), tidal volume (VT) and PEEP values, clinical findings and treatment applications were retrospectively obtained from the hospital database for both groups. In this way, ventilator setting parameters (PIP, PEEP, ΔP, Pplt, VT), c-reactive protein (CRP), procalcitonin, D-dimer, lactate dehydrogenase (LDH), neutrophil/lymphocyte ratio, ferritin, urea, creatinine, platelets, highest and lowest values for lymphocyte measurements for the period when patients in the group not developing barotrauma (NBG) were intubated and for the period until barotrauma developed in patients in the barotrauma (BG) group were recorded for statistical analysis. Similarly, cumulative steroid doses in intensive care, lowest pO_2_/FiO_2_ values and total duration in prone position were calculated for the groups (until barotrauma developed in the BG group).

### Statistical analysis

All statistical analyses in the study were completed using IBM SPSS Statistics 25 and InStat3 GraphPad Statistics Software. Nonparametric statistical analyses were used as the subject numbers in one group were lower than 30. The Mann−Whitney U test was used to compare the exitus and discharge groups. Chi-square test was used to compare the category data of the groups. Analysis results were accepted as being significantly different if *P* < 0.05.

## Results

In the date interval when data were collected in the study, a total of 96 patients in the ICU had confirmed COVID-19 diagnosis with RT-PCR tests based on nasopharyngeal swabs or endotracheal aspirate samples. Twenty-one patients who did not abide by the inclusion criteria were excluded from the study. Of the 75 patients included in the study, 10 were identified to have ventilator-related barotrauma (13%). All patients included in the study were assessed to have common findings of shortness of breath along with ground glass appearance with subpleural localisation on thoracic HRCT. However, no patient was observed to have any bullae, blebs or cyst-like pulmonary lesions which may cause PX, haemothorax or pneumomediastinum on initial radiological images.

**Fig. 2.**
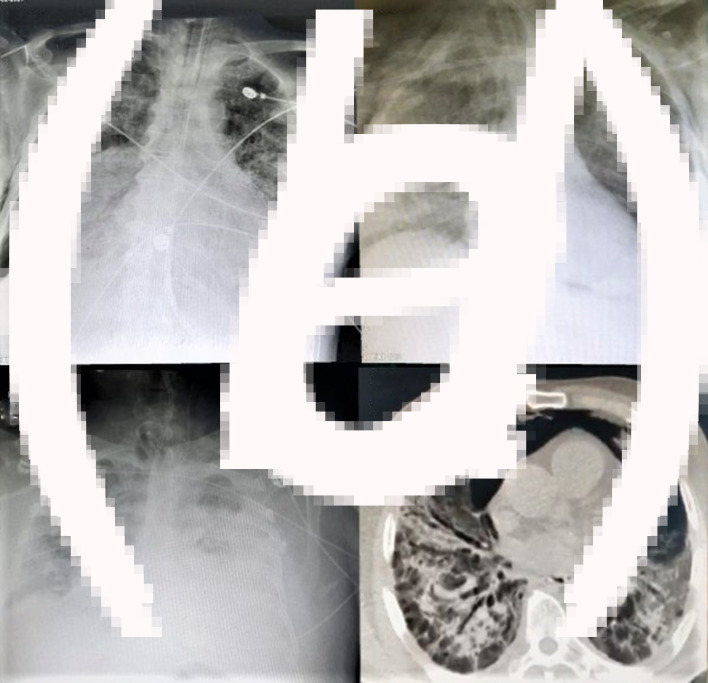
Pneumothorax – right side (a), pneumothorax – left side (b), haemothorax – left side (c), pneumomediastinum (d).

Of the patients who received invasive MV support, unilateral PX in five cases, bilateral PX in two cases, haemothorax in one case and pneumomediastinum with subcutaneous emphysema in two cases occurred (Table 1) (shown in Fig. 2). Both patients with bilateral PX were tracheotomised and PX was observed to develop immediately after the RM procedure. PX developed at the same time in one of the cases with bilateral PX, while in the other one PX first developed on the right and one day later on the left. It was noticed that subcutaneous emphysema developed after returning to the supine position in two patients who underwent RM in the prone position, and then immediately thoracic HRCT images were taken and the presence of simultaneous pneumomediastinum was identified. Spontaneous regression was observed in these two cases without any intervention required. Diagnosis of the case developing haemothorax was made with the presence of pleural fluid on routine chest radiography and sudden fall in haematocrit values. All cases developing haemothorax and PX had chest tube drainage (tube thoracostomy) for 5–7 days duration for treatment purposes.

**Table 1.** Demographics and clinical characteristics of the barotrauma group

Nature of barotrauma	Location	*N*	PCB	Comorbidity	TSB (day)	Gender	Age	Outcome
Pneumothorax	Right	3	IMV/IMV/ RM	CAD + HT + DM / HT / -	14 /23 / 25	F / M / M	80 / 68 / 49	EX /EX / Discharged
	Left	2	IMV/IMV	HT /CAD + DM	26 / 20	M / M	86 /59	Discharged / Discharged
	Bilateral	2	RM /RM	DM /none	29 /23	F / F	60 / 42	EX/Discharged
PMSE		2	RM /IMV	HT /none	17/24	M / F	53 / 36	EX/Discharged
Haemothorax	Left	1	IM, AM	none	19	M	44	Discharged

PMSE: pneumomediastinum with subcutaneous emphysema, PP: prone position, AM: anticoagulant medication, RM: recruitment manoeuver, CAD: coronary artery disease, HT: hypertension, DM: diabetes mellitus, IMV: invasive mechanical ventilation, *N*: number of patients, PCB: possible cause of barotrauma TSB: time between symptom onset and barotrauma.

Statistical comparisons between the groups did not identify any significant difference in terms of demographic characteristics; however, the barotrauma group (BG) had longer duration of intensive care stay, duration in prone position and total MV duration at statistically significant levels (*P* < 0.05) (Table 2). There was no significant difference between the two groups in terms of ventilator setting parameters such as PIP, VT, Pplt and ΔP. But barotrauma cases had higher max PEEP levels identified compared to those without barotrauma (*P* < 0.05) (Table 3). Contrary to this, those with barotrauma had lower min pO_2_/FiO_2_ levels compared to those without barotrauma (*P* < 0.05) (Table 4).

**Table 2.** Demographic and clinical characteristics of the study patients and comparison between groups

	All patients	Barotrauma group (BG)	Non-barotrauma group (NBG)	*P values
***n***	75	10	65	–
**Female,** (**%)**	24(32)	4(40)	20(31)	0.6338
**Age, year**	60 ± 17.9	59 ± 19.6	60 ± 17.9	0.8831
**Smoking,** (**%)**	42(56)	5(50)	37(61)	0.8738
**ICULOS, days**	20.6 ± 13.2	36.0 ± 16.4	18.6 ± 11.4	0.0042**
**Comorbidity,** (**%)**	44(59)	6(60)	38(58)	0.7508
**Prone position,** (**%)**	51(68)	10(100)	41(63)	–
**DTPP, hour**	51.5 ± 48.3	90.4 ± 57.8	46.5 ± 45.1	0.0197**
**Tracheostomy,** (**%)**	11(15)	4(40)	7(11)	0.0724
**DMV, day**	14.3 ± 11.0	28.9 ± 12.2	12.4 ± 9.5	0.0009**
**RecA,** (**%)**	55(73)	10(100)	45(70)	–
**CSD, mg**	850 ± 563	703 ± 239	869 ± 591	0.5793
**TocTr,** (**%)**	17(23)	3(30)	14 (22)	0.7940
**PlasTr,** (**%)**	16(21)	4(40)	12(18)	0.6174

* *P* values were calculated by the Mann−Whitney test. The values belonging to the groups are given as mean ± standard deviation. ICULOS: intensive care unit length of stay, DTPP: duration of time in prone position, DMV: duration of mechanical ventilation, RecA: recruitment application, CSD: cumulative steroid dosage, TocTr: tocilizumab treatment, PlasTr: plasmapheresis treatment, ** *P* < 0.05.

**Table 3.** Comparison of groups in terms of ventilator parameters

	Barotrauma group (BG)	Non-barotrauma group (NBG)	*P values
**PIP, cmH_2_O**	34.8 ± 4.7	35.8 ± 4.8	0.5177
**ΔP, cmH_2_O**	23.3 ± 3.7	23.9 ± 4.606	0.7731
**Pplt, cmH_2_O**	30.0 ± 3.6	30.7 ± 3.5	0.6625
**VT, ml/kg**	6.7 ± 1.4	7.0 ± 1.3	0.3619
**Max PEEP, cmH_2_O**	15.3 ± 1.1	13.6 ± 1.7	0.0150**

* *P* values were calculated by the Mann−Whitney test. Values are given as mean ± standard deviation, PIP: peak inspiratory pressure, ΔP: driving pressure, Pplt: plateau pressure, VT: tidal volume (ml/kg) PEEP: positive end-expiratory pressure, Max: maximum, ** *P* < 0.05.

**Table 4.** Some haematological and biochemical analysis results of the study patients and comparison between groups

	All patients	Barotrauma group (BG)	Non-barotrauma group (NBG)	*P values
***n***	75	10	65	–
**Min pO_2_/ FiO_2_, ratio**	84.6 ± 52.5	49.4 ± 9.6	89.1 ± 54.1	0.0325**
**Max urea, mg/dl**	94.5 ± 52.5	119.1 ± 60.2	91.3 ± 51.2	0.2439
**Max creatinine, mg/dl**	1.58 ± 1.11	1.43 ± 0.81	1.60 ± 1.14	0.7428
**CRP, mg/l**	179 ± 70	222 ± 66	174 ± 69	0.0715
**Procalcitonin, ng/ml**	6.8 ± 14.6	6.8 ± 9.4	6.84 ± 15.18	0.9912
**D-Dimer, ng/ml**	5454 ± 7181	6541 ± 4714	5313 ± 7462	0.0609
**Ferritin, ng/ml**	2422 ± 4513	4305 ± 6873	2178 ± 4145	0.1068
**Peak LDH, U/l**	1237 ± 1620	2174 ± 2659	1115 ± 1429	0.0441**
**Min LC, (×10^3^/μl)**	0.68 ± 0.65	0.46 ± 0.27	0.71 ± 0.68	0.2485
**Plt count, (×10^5^/μl)**	334 ± 136	264 ± 129	343 ± 136	0.1899
**WBC, (×10^3^/μl)**	16.7 ± 8.2	19.5 ± 7.6	16.3 ± 8.2	0.2691
**NLR**	38.4 ± 40.1	65.1 ± 69.2	35.0 ± 34.3	0.2579

* *P* values were calculated by the Mann−Whitney test. The values belonging to the groups are given as mean ± standard deviation. Max: maximum, Min: minimum, CRP: C reactive protein, LC: min lymphocyte count, NLR: neutrophil/lymphocyte ratio, LDH: lactate dehydrogenase, Plt: platelet, ** *P* < 0.05.

Patients in the BG group had statistically significantly higher peak lactate dehydrogenase (LDH) values compared to the group without barotrauma (NBG) (*P* < 0.05) (Table 4). Other parameters shown in Tables 2 and 4 were not identified to have statistical differences between those with barotrauma and those without barotrauma (*P* > 0.05). Though not statistically significant, it is notable that the BG group had slightly higher CRP, D-dimer and ferritin values. According to the chi-square test, exitus or discharge status had a close relationship with disease comorbidity but no relationship with smoking, prone position, barotrauma, tracheostomy, RM administration, tocilizumab and convalescent treatment (Table 5).

**Table 5.** Comparison of risk factors of discharged and exitus patients with chi-square test

	Value	df	Asymptotic significance (two sided)	Exact significance (two sided)
**Comorbidity**	9.784^a^^,^^b^	1	0.002	
**Smoking**	0.166^a^^,^^c^	1	0.684	
**Prone position**	2.939^a^^,^^d^	1	0.086	
**Barotrauma**	0.249^a^^,^^e^	1	0.618	0.683^z^
**Tracheostomy**	0.362^a^^,^^f^	1	0.547	0.703^z^
**Recruitment (RM)**	0.697^a^^,^^g^	1	0.404	0.479^z^
**TocTr**	0.276^a^^,^^h^	1	0.599	0.753^z^
**PlasTr**	0.022^a^^,^^i^	1	0.881	1.000^z^

a Pearson's chi-square, *z* Fisher's exact test, *y* continuity correction.

b 0 cells (0.0%) have expected count less than 5. The minimum expected count is 7.57.

c 0 cells (0.0%) have expected count less than 5. The minimum expected count is 8.26.

d 0 cells (0.0%) have expected count less than 5. The minimum expected count is 5.85.

e 2 cells (50.0%) have expected count less than 5. The minimum expected count is 2.41.

f 1 cells (25.0%) have expected count less than 5. The minimum expected count is 2.75.

g 1 cells (25.0%) have expected count less than 5. The minimum expected count is 3.10.

h 1 cells (25.0%) have expected count less than 5. The minimum expected count is 4.82.

i 1 cells (25.0%) have expected count less than 5. The minimum expected count is 3.79.

## Discussion

**Fig. 3.**
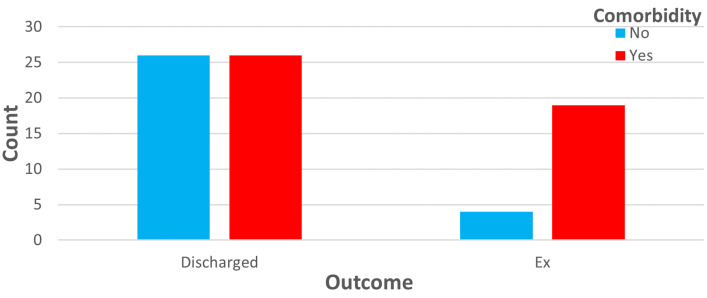
Comorbidity rates of patients who were exitus or discharged.

While the majority of COVID-19 patients experience the disease with mild symptoms, 5–12% of cases are monitored in intensive care units with diagnoses like ARDS or multiorgan failure [8,9]. Mortality for COVID-19 ARDS patients is reported as nearly 50% (12–78%) [10,11]. Despite the same vector (SARS-CoV-2) in the aetiology, outcomes may display variability. Severity of infection, physiological features of patients, comorbidities and personal ventilation response against hypoxaemia play important roles in this variability [12]. For COVID-19 ARDS patients monitored in our intensive care unit, total mortality rates were found to be 30% (29/96). Among these patients, the mortality rate was 40% (4/10) in the BG group and 29% (19/65) in the NBG group. In spite of higher mortality rates in the group developing barotrauma, the difference was not statistically significant. However, the mortality rates of patients with comorbidities were higher and this was statistically significant. In other words, the presence of comorbidity in patients can be easily said to be an independent risk factor for COVID-19 (shown in Fig. 3).

The lack of known available treatment for the disease has led to many pharmacological agents (hydroxychloroquine, azithromycin, tocilizumab, anakinra, oseltamivir, favipiravir, lopinavir/ritonavir) being trialled and some vaccination studies were completed and some are ongoing [13,14]. At this stage, supportive treatments such as hydration, agents strengthening the immune system, convalescent plasma and high concentration oxygen therapies or MV are still important for those who suffer from the disease. In line with our COVID-19 ARDS treatment protocol, all patients with SARS-CoV-2 infection are administered favipiravir, hydroxychloroquine, azithromycin and high-dose (30 g/day, 7 days) ascorbic acid from the first day as standard [15]. Additionally, 17 cases developing macrophage activation syndrome (MAS) were administered tocilizumab (IL-6 antagonist) and 16 cases meeting the criteria published in the national guidelines for COVID-19 treatment in adult patients received convalescent (immune) plasma [16].

The incidence of barotrauma among ARDS patients following MV treatment is reported to be about 6.5%, but this rate is higher for ARDS caused by members of the coronavirus family [3, 17, 18]. Reaching rates of 12% during the SARS epidemic and 30% durıng the MERS epidemic, some recent research reported barotrauma at rates of 15–40% during the COVID-19 pandemic [17−19]. In our study, our barotrauma incidence rates were 13% for ARDS patients on MV support. This low barotrauma incidence rate compared with the literature may be linked to implementing MV in accordance with the ARDSnet ventilation strategy protocols in our intensive care. However, this rate (13%) is still higher than barotrauma rates in ARDS forming due to non-coronavirus vectors. One of the most important reasons for this is that ARDS with COVID-19 disease is accompanied by a tableau with diffuse alveolar injury, different to classic ARDS [18]. In pneumonia caused by SARS-CoV-2, injury to alveoli is accompanied by fibrin microthrombus within the vascular area and there are even articles reporting pulmonary cysts form in advanced stages [19]. Supporting this, in recent times cases are mentioned where spontaneous pneumomediastinum or PX develops in patients with a COVID-19 background who did not receive MV support [20-21]. In the large COVID-19 case series multi-centre retrospective study by Martinelli *et al*. [22], 32% of cases (non-ventilated and non-intubated) were reported to have PX. According to this and previous reports, inflammation, consolidation and necrosis occurring in pulmonary parenchyma during COVID-19 disease leads to the formation of cystic and cavitary lesions in the lungs over time. In fact, due to the increased fistulation risk between parenchyma and pleura, spontaneous PX may be triggered and it is reported that a single mechanism cannot explain this situation. Similarly, cases developing spontaneous pneumomediastinum and PX without receiving MC support were reported in the SARS-CoV-1 infection observed in 2003 [23]. The probable reason for this was shown to be ischaemic parenchymal injury, pulmonary fibrosis, low lung compliance and formation of inflammatory exudate in the airway [20–24]. As a result, barotrauma can develop not only in mechanically ventilated patients but also in non-ventilated patients with COVID-19. In our patients with non-invasive MV support (N:8) spontaneous pneumomediastinum or PX linked to SARS-CoV-2 infection was not observed.

In spite of MV being the most important supportive treatment in intensive care, it may be difficult to provide sufficient oxygenation for severe ARDS cases. Gattinoni *et al*. identified two different phenotypes which interact with each other in pneumonia caused by SARS-CoV-2 (L and H types). Type-L COVID-19 pneumonia involves vasoplegia as the basis of hypoxaemia, with low elasticity and low pulmonary weight and non-invasive ventilation and high-flow oxygen therapy may be sufficient [12]. According to this description, eight patients included in our study had type-L pneumonia and could be treated with non-invasive oxygen support without progressing to type-H. In the advanced stages of the disease, increased interstitial pulmonary oedema due to the effect of negative intrathoracic pressure and inflammation cause an increase in pulmonary weight and elasticity which may increase the amount of unventilated pulmonary tissue (type-H COVID-19 pneumonia). In this situation, to correct hypoxaemia the RM is needed and to open the atelectatic lung areas it is necessary to continuously increase the patient's airway pressure for a certain period. This may be performed with methods such as PEEP, CPAP, pressure-controlled MV, sigh manoeuvre, spontaneous respiration, placing the patient in prone position and high-frequency ventilation [6, 25]. However, none of the implementations are innocuous and they may cause formation of barotrauma in the lungs. In our cases, a total of 51 patients with pO_2_/FiO_2_ ratio <150 were placed in prone position accompanied by continuous muscle relaxants and sedation for 16–24 h and all had good responses to the first implementation. One patient was observed to have severe hypotension related to prone position, while 14 patients were observed to have second-stage compression wounds on the skin at the temporal and mandibular bone protrusions. In our comparisons between the groups, BG patients had longer duration in prone position and in parallel with this, the same group of patients were identified to have longer MV durations and intensive care durations. Though previous studies stated that prone position is protective against ventilator-related barotrauma risk, an increase in barotrauma risk was observed in our cases [26]. This situation may be linked to the low number in the sample and the longer duration in prone position. Alveolar rupture may occur as a result of widespread alveolar injury in COVID-19 patients, and this situation may cause interstitial emphysema. The air in interstitial emphysema causes dissection along the bronchovascular sheath toward the mediastinum over time leading to pneumomediastinum or may progress in some patients to cause PX or subcutaneous emphysema [19].

In our total of 55 patients who responded to PEEP increase and were haemodynamically stable, RM was applied 2 times per day by keeping the airway pressure at 30 cmH_2_O for 60 s in CPAP mode. However, as RM was performed in those with barotrauma, the relationship between RM and the presence of barotrauma could not be researched (Table 2). This situation is a limitation of our study. Additionally, two tracheostomy patients with RM applied developed bilateral PX. This situation leads to consideration that tracheostomy and RM implementation may cause an increased risk of bilateral PX. There is a need for more comprehensive research to explain this topic.

During MV in our intensive care, PEEP values for patients were set according to the ARDSnet lower PEEP/higher FiO_2_ table. Accordingly, PEEP values increased in direct proportion to FiO_2_. In our research, patients with BG had worse oxygenation and were identified to have higher PEEP values and lower min pO_2_/FiO_2_ ratio compared to the NBG group during MV (Tables 3 and 4). Accordingly, as with Eisner *et al*. [27] the inference may be made that higher PEEP values are associated with increased barotrauma risk. However, ventilator setting parameters such as PIP, ΔP, VT and Pplt were similar between the groups. Whether barotrauma development during invasive MV is related to ventilator setting parameters is still a controversial issue. Study findings by Weg *et al*. [28] showed that barotrauma development was not associated with ventilator airway pressure and tidal volumes, similar to our study. In our comparison taking peak values for LDH levels, a biochemical marker of cellular damage in the lungs, significantly higher values were identified in the BG group (*P* = 0.0441). Chu *et al*. identified a significant correlation between elevated LDH values and spontaneous pneumomediastinum (SP) in a study of SARS [23]. According to this result, it is not wrong to say that in addition to damage caused by SARS-CoV-2 in lung tissue, pressurised ventilation applied to the lungs during MV may affect barotrauma formation.

As the duration of intubation increases, the complication risk is known to increase. If a non-COVID-19 patient with invasive MV support cannot be weaned from the ventilator within 7–10 days, the percutaneous tracheostomy procedure is recommended in order to perform procedures such as bronchial cleaning, feeding, patient mobilisation and nursing care more easily [29]. Thus, the patient's airway resistance and respiratory work reduces easing weaning from the ventilator. However, it is recommended to wait until 21 days for COVID-19 patients due to the reduction in virus load in COVID-19 patients with each day. Current data show that less than 10% of COVID patients in intensive care have tracheostomy [30]. In our intensive care, 11 patients (11%) had percutaneous dilatational tracheostomy procedure performed at mean 23 ± 3 days of intubation. Apart from two (EX), all patients with tracheostomy had tracheostomies closed and were discharged from hospital. In our study, there was no statistical significance between tracheostomy and barotrauma; however, the low *P* value for the statistical significance between tracheostomy and barotrauma (*P* = 0.0724) leads to consideration that a significant relationship may occur between tracheostomy and barotrauma risk in research with study groups containing larger numbers.

Haemothorax generally forms due to thoracic trauma, while it rarely develops for nontraumatic (spontaneous) reasons. Among these vascular pathologies, necrotising infections, connective tissue diseases, pleural diseases, endometriosis, neoplasia and haemorrhage disorders may be listed [31]. During monitoring and treatment with MV for severe ARDS tableau, a young patient with no chronic disorder was identified to have haemothorax on the 19th day after symptom onset and a total of 2100 ml blood was drained from the pleural cavity with tube thoracostomy. When the cause of haemothorax in the patient was researched, there was no risk factor found, apart from LMWH (enoxaparin 0.6 mg/kg 2X1 subcutaneous) begun at therapeutic dose with the aim of protecting against hypercoagulability complications. The patient's anti-FXa levels were not measured. However, no clinical finding (like haematuria, haematochezia, melena and haemorrhagic tracheal secretions) of haemorrhage that may form due to high anticoagulant treatment was observed. When this situation is assessed in light of the literature, this patient monitored with invasive MV for a long duration (>12 days) was considered to have separation of adhesions between the pleura leaves forming due to the large amount of variation in pleural pressure with the advance of subpleural localised infection and that haemothorax may be due to this [31, 32].

Barotrauma findings observed in our patients emerged 22 ± 3.6 days after symptoms began. Chu *et al*. reported SP was observed 19.6 ± 4.6 days later in SARS patients. The place for steroid treatment in viral-vector ARDS is still controversial, and we administered low-dose methylprednisolone to patients considered to have interstitial pulmonary oedema with pO_2_/FiO_2_ <150. Our comparison in terms of cumulative steroid dose did not identify a significant difference between the two groups. This result complies with the study by Chu *et al*. about SARS patients [23].

## Conclusion

In COVID-19 ARDS patients, alveolar injury caused by the infection with the contribution of MV may cause more frequent barotrauma compared to classic ARDS and this situation significantly increases the patients' duration on MV and in intensive care. In terms of reducing mortality and morbidity in these patients, MV treatment should be carefully maintained within the framework of lung protecting strategies and complications should be identified early and treated.

## Data Availability

The datasets used and analysed during the current study are available from the corresponding author on reasonable request.

## References

[1] Özlü A and Öztaş D (2020) Learning lessons from the past in combating the novel coronavirus (Covid-19) pandemic. Ankara Medical Journal 20, 468–481.

[2] Ranieri VM (2012) Acute respiratory distress syndrome: the Berlin definition. Journal of the American Medical Association 307, 2526–2533. 22797452 10.1001/jama.2012.5669

[3] Anzueto A (2004) Incidence, risk factors and outcome of barotrauma in mechanically ventilated patients. Intensive Care Medicine 30, 612–619. 14991090 10.1007/s00134-004-2187-7

[4] De Lassence A (2006) Pneumothorax in the intensive care unit: incidence, risk factors, and outcome. Anesthesiology 104(1), 5–13. doi: 10.1097/00000542-200601000-00003. 16394682

[5] Thompson BT and Bernard GR (2011) ARDS Network (NHLBI) studies: successes and challenges in ARDS clinical research. Critical Care Clinics 27, 459–468. 21742211 10.1016/j.ccc.2011.05.011 PMC3143063

[6] Cavalcanti AB (2017) Effect of lung recruitment and titrated positive end-expiratory pressure (PEEP) vs low PEEP on mortality in patients with acute respiratory distress syndrome − A randomized clinical trial. Journal of the American Medical Association 318(14), 1335–1345. doi: 10.1001/jama.2017.14171. 28973363 PMC5710484

[7] Meade MO (2008) Ventilation strategy using low tidal volumes, recruitment maneuvers, and high positive end-expiratory pressure for acute lung injury and acute respiratory distress syndrome: a randomized controlled trial. Journal of the American Medical Association 299(6), 637–645. doi: 10.1001/jama.299.6.637. 18270352

[8] Livingston E and Bucher K (2020) Coronavirus disease 2019 (COVID-19) in Italy. Journal of the American Medical Association 323, 1335. 32181795 10.1001/jama.2020.4344

[9] Grasselli G, Pesenti A and Cecconi M (2020) Critical care utilization for the COVID-19 outbreak in Lombardy, Italy: early experience and forecast during an emergency response. Journal of the American Medical Association 323, 1545–1546. 32167538 10.1001/jama.2020.4031

[10] Yang X (2020) Clinical course and outcomes of critically ill patients with SARS-CoV-2 pneumonia in Wuhan, China: a single-centered, retrospective, observational study. Lancet Respiratory Medicine 8(5), 475–481. doi: 10.1016/S2213-2600(20)30079-5. 32105632 PMC7102538

[11] Eastin C, Eastin T (2020) Characteristics and outcomes of 21 critically ill patients with COVID-19 in Washington State: Arentz M, Yim E, Klaff L *et al*. Journal of the American Medical Association 323(16), 1612–1614. doi: 10.1016/j.jemermed.2020.04.002. 32191259 PMC7082763

[12] Gattinoni L (2020) COVID-19 pneumonia: different respiratory treatments for different phenotypes? Intensive Care Medicine 46, 1099–1102. 32291463 10.1007/s00134-020-06033-2 PMC7154064

[13] Zhang L and Liu Y (2020) Potential interventions for novel coronavirus in China: a systematic review. Journal of Medical Virology 92, 479–490. 32052466 10.1002/jmv.25707 PMC7166986

[14] Cavalli G (2020) Interleukin-1 blockade with high-dose anakinra in patients with COVID-19, acute respiratory distress syndrome, and hyperinflammation: a retrospective cohort study. Lancet Rheumatology 2(6), e325–e331. doi: 10.1016/S2665-9913(20)30127-2. 32501454 PMC7252085

[15] Douedi S and Miskoff J (2020) Novel coronavirus 2019 (COVID-19): a case report and review of treatments. Medicine *(*Baltimore*)*. 99(19), e20207. Available at https://journals.lww.com/md-journal/Fulltext/2020/05080/Novel_coronavirus_2019__COVID_19___A_case_report.93.aspx. 32384516 10.1097/MD.0000000000020207 PMC7220032

[16] Duan K (2020) Effectiveness of convalescent plasma therapy in severe COVID-19 patients. Proceedings of the National Academy of Sciences 117, 9490 LP–9496. 10.1073/pnas.2004168117 PMC7196837 32253318

[17] Kao H-K (2005) Pneumothorax and mortality in the mechanically ventilated SARS patients: a prospective clinical study. Critical Care 9, R440–R445. 16137358 10.1186/cc3736 PMC1269458

[18] McGuinness G (2020) Increased incidence of barotrauma in patients with COVID-19 infection on invasive mechanical ventilation. Radiology 297(2), e252–e262. doi: 10.1148/radiol.2020202352. 32614258 PMC7336751

[19] Liu K (2020) COVID-19 with cystic features on computed tomography: a case report. Medicine *(*Baltimore*)*. 99(18), e20175. Available at https://journals.lww.com/md-journal/Fulltext/2020/05010/COVID_19_with_cystic_features_on_computed.68.aspx. 32358406 10.1097/MD.0000000000020175 PMC7440163

[20] Zhou C (2020) COVID-19 with spontaneous pneumomediastinum. The Lancet Infectious Diseases 20, 510. 32164830 10.1016/S1473-3099(20)30156-0 PMC7128610

[21] Sun R, Liu H and Wang X (2020) Mediastinal emphysema, giant bulla, and pneumothorax developed during the course of COVID-19 pneumonia. Korean Journal of Radiology 21, 541–544. 32207255 10.3348/kjr.2020.0180 PMC7183834

[22] Martinelli AW (2020) COVID-19 and pneumothorax: a multicentre retrospective case series. European Respiratory Journal 56, 2002697. 10.1183/13993003.02697-2020 PMC7487269 32907891

[23] Chu CM (2004) Spontaneous pneumomediastinum in patients with severe acute respiratory syndrome. European Respiratory Journal 23, 802 LP–804. 10.1183/09031936.04.00096404 15218989

[24] Desai SR (2002) Acute respiratory distress syndrome: imaging of the injured lung. Clinical Radiology 57, 8–17. 11798197 10.1053/crad.2001.0889

[25] Hess DR (2015) Recruitment maneuvers and PEEP titration. Respiratory Care 60, 1688 LP–1704. 26493593 10.4187/respcare.04409

[26] Kopterides P, Siempos II and Armaganidis A (2009) Prone positioning in hypoxemic respiratory failure: meta-analysis of randomized controlled trials. Journal of Critical Care 24, 89–100. 19272544 10.1016/j.jcrc.2007.12.014

[27] Eisner MD (2002) Airway pressures and early barotrauma in patients with acute lung injury and acute respiratory distress syndrome. American Journal of Respiratory and Critical Care Medicine 165, 978–982. 11934725 10.1164/ajrccm.165.7.2109059

[28] Weg JG (1998) The relation of pneumothorax and other air leaks to mortality in the acute respiratory distress syndrome. The New England Journal of Medicine 338, 341–346. 9449726 10.1056/NEJM199802053380601

[29] Lin MC (1999) Pulmonary mechanics in patients with prolonged mechanical ventilation requiring tracheostomy. Anaesthesia and Intensive Care 27, 581–585. 10631410 10.1177/0310057X9902700604

[30] Miles BA (2020) Tracheostomy during SARS-CoV-2 pandemic: recommendations from the New York Head and Neck Society. Head & Neck 42, 1282–1290. 32304119 10.1002/hed.26166 PMC7264578

[31] Janik M (2014) Non-traumatic and spontaneous hemothorax in the setting of forensic medical examination: a systematic literature survey. Forensic Science International 236, 22–29. 24529771 10.1016/j.forsciint.2013.12.013

[32] Singh S, (2009) Idiopathic massive spontaneous hemothorax: adhesion disruption. World Journal of Surgery 33, 489–491. 19123028 10.1007/s00268-008-9844-x

